# Anthropometry in 5- to 9-Year-Old Greenlandic and Ukrainian Children in Relation to Prenatal Exposure to Perfluorinated Alkyl Substances

**DOI:** 10.1289/ehp.1408881

**Published:** 2015-03-26

**Authors:** Birgit Bjerre Høyer, Cecilia Høst Ramlau-Hansen, Martine Vrijheid, Damaskini Valvi, Henning Sloth Pedersen, Valentyna Zviezdai, Bo A.G. Jönsson, Christian H. Lindh, Jens Peter Bonde, Gunnar Toft

**Affiliations:** 1 Danish Ramazzini Centre, Department of Occupational Medicine, Aarhus University Hospital, Aarhus, Denmark; 2 Section for Epidemiology, Department of Public Health, Aarhus University, Aarhus, Denmark; 3 Centre for Research in Environmental Epidemiology (CREAL), Barcelona, Spain; 4 Pompeu Fabra University, Barcelona, Spain; 5 CIBER de Epidemiología y Salud Pública (CIBERESP), Spain; 6 Primary Health Care Clinic, Nuuk, Greenland; 7 Department of Social Medicine and Organization of Public Health, Kharkiv National Medical University, Kharkiv, Ukraine; 8 Division of Occupational and Environmental Medicine, Lund University, Lund, Sweden; 9 Department of Occupational and Environmental Medicine, Copenhagen University Hospital Bispebjerg, Copenhagen, Denmark

## Abstract

**Background:**

In some animal studies, perfluorinated alkyl substances are suggested to induce weight gain. Human epidemiological studies investigating these associations are sparse.

**Objective:**

We examined pregnancy serum concentrations of perfluorooctanoate (PFOA) and perfluorooctane sulfonate (PFOS) and the prevalence of offspring overweight (> 1 SD) and waist-to-height ratio (WHtR) > 0.5 at 5–9 years of age.

**Methods:**

Sera from 1,022 pregnant women enrolled in the INUENDO cohort (2002–2004) from Greenland and Kharkiv (Ukraine) were analyzed for PFOA and PFOS using liquid chromatography–tandem mass spectrometry. Relative risks (RR) of being overweight and having WHtR > 0.5 in relation to continuous and categorized (tertiles) PFOA and PFOS were calculated at follow-up (2010–2012) using generalized linear models.

**Results:**

Pooled PFOA median (range) was 1.3 (0.2–5.1) and PFOS median (range) was 10.8 (0.8–73.0) ng/mL. For each natural logarithm-unit (ln-unit) increase of pregnancy PFOA, the adjusted RR of offspring overweight was 1.11 [95% confidence interval (CI): 0.82, 1.53] in Greenlandic children. In Ukrainian children, the adjusted RR of offspring overweight was 1.02 (95% CI: 0.72, 1.44) for each ln-unit increase of pregnancy PFOA. Prenatal exposure to PFOS was not associated with overweight in country-specific or pooled analysis. The adjusted RR of having WHtR > 0.5 for each ln-unit increase of prenatal exposure to PFOA was 1.30 (95% CI: 0.97, 1.74) in the pooled analysis. For 1–ln-unit increase of prenatal exposure to PFOS, the adjusted RR of having a WHtR > 0.5 was 1.38 (95% CI: 1.05, 1.82) in the pooled analysis.

**Conclusions:**

The results indicate that prenatal PFOA and PFOS exposures may be associated with child waist-to-height ratio > 0.5. Prenatal PFOA and PFOS exposures were not associated with overweight.

**Citation:**

Høyer BB, Ramlau-Hansen CH, Vrijheid M, Valvi D, Pedersen HS, Zviezdai V, Jönsson BA, Lindh CH, Bonde JP, Toft G. 2015. Anthropometry in 5- to 9-year-old Greenlandic and Ukrainian children in relation to prenatal exposure to perfluorinated alkyl substances. Environ Health Perspect 123:841–846; http://dx.doi.org/10.1289/ehp.1408881

## Introduction

Perfluorinated alkyl substances (PFAS) have been used extensively in various consumer products such as textiles, leather, paper, and food wrapping because of their water-, dirt-, and oil-repellent properties (Fromme et al. 2009). A phaseout of the production of perfluorooctanoate (PFOA) and perfluorooctane sulfonate (PFOS) was initiated in 2000 by the major U.S. producers (U.S. Environmental Protection Agency 2010). However, because both compounds have half-lives of several years in humans (Olsen et al. 2007) and precursor substances are able to transform into PFOA and PFOS, as reviewed by Fromme et al. (2009), we are still being exposed through diet, packaged food, drinking water, and dust.

The PFAS bind to albumin in blood (D’eon et al. 2010), and they are able to cross placenta (Midasch et al. 2007). *In vivo* and *in vitro* studies suggest that PFOA and PFOS are endocrine disruptors (Du et al. 2013a, 2013b). An animal study reported that prenatal exposure to PFOA was associated with overweight and elevated serum leptin and insulin in young adult female mice (Hines et al. 2009), and a recent study reported no association in mice (Ngo et al. 2014). In humans, *in utero* exposure to PFOA has been associated with lower birth weight (Fei et al. 2007, 2008) and smaller size at birth (Apelberg et al. 2007; Chen et al. 2012), although only modestly so. Also, an inverse association between prenatal exposure to PFOA and PFOS and weight and BMI has been reported among infant boys (Andersen et al. 2010); and in a recent study, prenatal PFOA exposure was associated with obesity in adult females (Halldorsson et al. 2012).

To our knowledge, only one study has been performed in school-age children, finding no association between *in utero* PFOA and PFOS exposure and overweight, body mass index (BMI), and waist circumference (Andersen et al. 2013). Thus, the aim of this study was to investigate the association between prenatal exposure to PFOA and PFOS and subsequent anthropometry in the offspring at 5–9 years in European and Arctic birth cohorts.

## Methods

*Study population and data collection*. A total of 1,183 pregnant women from Greenland (*n* = 571) and Kharkiv (Ukraine) (*n* = 612) were enrolled in the birth cohorts throughout pregnancy from antenatal health care clinics. The women provided a blood sample at a mean (± SD) gestational age of 24 ± 10 weeks, during the period from May 2002 to February 2004. To be eligible for the study, the woman had to be born in the country of study, be pregnant, and be at least 18 years of age. Further details on the baseline study population are available elsewhere (Toft et al. 2005). A follow-up was conducted from January 2010 through May 2012 when the children were between 5 and 9 years old [median (10th–90th percentile), 8.3 (7.3–9.1) in Greenland and 7.0 (6.6–7.6)] in Ukraine (Høyer et al. 2014). Parents or guardians responded to questions concerning lifestyle and other characteristics in a face-to-face interview or by filling in a questionnaire themselves.

A total of 1,023 mother–child pairs (singleton births) had available blood samples and were followed up. One Ukrainian child was excluded due to an extreme BMI value (7.4 kg/m^2^), leaving a study sample of 1,022 children. The study population was distributed between Greenland [*n* = 531 (52%)] and Ukraine [*n* = 491 (48%)].

The study was approved by local ethical committees: Ethical Committee for Human Research in Greenland (approval no. 2010–13) and the Commission on Ethics and Bioethics Kharkiv National Medical University in Ukraine (protocol number 7, 7 October 2009). All participating parents signed informed consent.

*Determination of PFOA and PFOS*. Plasma concentrations of PFOA and PFOS were analyzed at The Department of Occupational and Environmental Medicine in Lund, Sweden, using liquid chromatography–tandem mass spectrometry (LC/MS/MS). A detailed description of the method is presented elsewhere (Lindh et al. 2012). Briefly, aliquots of 100 μL serum were added to 25 μL of a water:acetonitrile (50:50) solution containing labeled internal standards. Proteins were precipitated with acetonitrile and shaken vigorously for 30 min. The samples were then centrifuged and the supernatant analyzed using an LC (UFLCXR; Shimadzu Corporation) connected to a hybrid triple quadrupole linear ion trap mass spectrometer (QTRAP 5500; AB Sciex). Limits of detection (LOD) were 0.04 ng/mL and 0.2 ng/mL for PFOA and PFOS, respectively, and all samples were above LOD. Coefficient of variation of duplicate samples worked up and analyzed on different days were 11% and 9% for PFOA and PFOS, respectively.

*Anthropometric measures*. The child’s weight was measured to the nearest 0.1 kg by a weighing scale available at the family’s home or at the clinics. In Greenland, the child’s height was measured with the child standing barefoot against a wall, marking the top of the head and measuring the height to the nearest centimetre by use of ordinary measuring tape. In Ukraine, standard stadiometers were used for height measuring. In Ukraine, all measurements were performed by the interviewer, and in Greenland, all measurements were performed by the interviewer except for those who were telephone-interviewed, in which case parents performed the measurements (*n* = 136 (25.6%). Child BMI was calculated from weight (kilograms)/height (meters squared). BMI was expressed as a sex- and age-specific *z-*score using the World Health Organization (WHO) Growth Standards 2007 (de Onis et al. 2007) to facilitate comparison of results with those shown in similar studies. Children with a BMI *z-*score > 1 SD were classified as overweight (de Onis et al. 2007).

Waist circumference was measured by measuring tape across abdomen corresponding to the umbilicus. Waist-to-height ratio (WHtR) was calculated from waist circumference (centimeters)/height (centimeters). A cut-off of WHtR > 0.5 indicated high risk WHtR and was based on earlier studies on children (Goulding et al. 2010; Maffeis et al. 2008; Mokha et al. 2010) because a WHtR > 0.5 has been associated with increased cardiometabolic morbidity in children (Mokha et al. 2010) and in adults (de Koning et al. 2007).

*Statistical analysis*. Characteristics of participants included in these analyses were compared with those of the participants who were lost to follow-up. Spearman’s rank correlation was used to assess the correlation between pregnancy levels of PFOA and PFOS. We used generalized additive models (GAM) to assess the shape of the relationship between pregnancy levels of PFOA and PFOS and BMI *z-*score, overweight, WHtR, and WHtR > 0.5. Because the GAMs did not always show linear association (*p*-values between 0.04 and 0.47), analyses were performed using both continuous exposures and using country-specific tertiles. Generalized linear models with robust variance estimation were used to estimate the relative risk (RR) of overweight and WHtR > 0.5 (Zou 2004). Multivariate linear regression models were used to assess the association between exposures and continuous BMI *z-*scores and WHtR. Pooled analyses were performed on continuous exposures in Greenlandic and Ukrainian children as no sign of heterogeneity was evident (exposures × country interaction, *p* > 0.20).

Potential confounders of the associations between prenatal PFAS exposures and the anthropometric outcomes were identified in literature: maternal age at birth, parity, pre-pregnancy BMI, maternal smoking during pregnancy, maternal alcohol consumption when trying to conceive, maternal education, duration of total breastfeeding, child age at follow-up, child sex, gestational age at blood sampling, birth weight, preterm birth, child sugary intake at follow-up, child fruit and vegetable intake at follow-up, and child physical activity level at follow-up. Directed acyclic graphs (DAGs) were used to select the confounders included in the final models using DAGitty software (Textor et al. 2011). All final multivariate models were adjusted for maternal age at birth (continuous, years), parity (dichotomous, 1, 2, > 2), maternal smoking during pregnancy (dichotomous, serum cotinine ≤ 10/> 10 ng/mL), maternal education (dichotomous, unskilled/skilled) and maternal prepregnancy BMI (continuous, kilograms per meter squared). The models of WHtR were additionally adjusted for child age (continuous, weeks) and sex. The pooled analysis was furthermore adjusted for country. Because missing data would cause a risk of selection bias as well as loss of power, missing covariate and outcome data were imputed using chained multiple imputation, generating 100 complete data sets (Donders et al. 2006; Sterne et al. 2009). We assumed that missing variables, including missing outcome variables, were missing at random, such that their values could be predicted by other variables (with known values) in the data set. Briefly, multiple different imputed datasets (*m =* 100) were created, and a set of random plausible values replaced each missing value, based on known subject characteristics and other predictors in the complete data set. This incorporated an appropriate variability across the data sets. The new 100 complete data sets were analyzed, producing a single set of results accounting for the variability of the missing data. The predictors of the model were country, PFOA, PFOS, gestational age at blood sampling, gestational age at birth, birth weight, total breastfeeding duration, maternal educational level, maternal age at birth, maternal cotinine level at blood sampling, maternal alcohol consumption when trying to conceive, maternal BMI, parity, child sex, child age at interview, child sugar intake at interview, child fruit and vegetable intake at interview, child physical activity level at interview, child BMI, child waist circumference, child hip circumference, child weight, child height, and child BMI *z-*score.

We evaluated effect modification of the association between the exposures and outcomes by sex and age using stratified analysis and by adding interaction terms in the model. Because birth weight may influence the risk of overweight later in life (Schellong et al. 2012), we restricted our analysis to children with normal birth weight (2,500–4,000 g; *n* = 370 in Greenland and *n* = 465 in Ukraine) in a sensitivity analysis. Timing of blood sampling may be related to POP levels and is possibly related to fetal adipose tissue development, so we adjusted for gestational age at blood sampling in a sensitivity analysis. Further, sugar intake (predominantly healthy vs. predominantly unhealthy; derived as sugary drinks or deserts < 6–7 vs. ≥ 6–7 times/week), fruit and vegetable intake (≤ 7 vs. > 7 times/week), and physical activity (< 2.75 vs. ≥ 2.75 hr/day) of the child at follow-up was added to the model as these are strongly related to the outcomes. Also, both exposures were added in the same model to allow mutual adjustment in the Greenlandic and Ukrainian populations. Further, we analyzed the association between PFAS and RR of overweight and WHtR > 0.5 in children with no missing data (complete cases). Finally, we explored the robustness of the imputation model by running the above-mentioned analysis on 20 and 150 imputed data sets. All statistical analysis was performed using STATA 13.1 (StataCorp, College Station, TX, USA), and the significance level was set at *p* < 0.05.

## Results

Levels of PFOA and PFOS in average and parental BMI did not differ between children who were not included in the present analysis due to loss to follow-up (*n* = 159) and those included in the study (*n* = 1,022) (see Supplemental Material, Table S1). Spearman’s rank correlation between PFOA and PFOS was 0.49 in Greenland and 0.51 in Ukraine. The characteristics of the study population have previously been presented in detail (Høyer et al. 2014). Briefly, compared with children from Greenland, children from Ukraine had lower birth weight, were younger at follow-up, and had a smaller BMI *z-*score at 5–9 years. Mothers from Ukraine had a lower prepregnancy BMI, were more often primiparous, and less often smokers than mothers from Greenland (Table 1). Data were more likely to be missing in Greenland than in Ukraine, and the most prevalent missing information was the outcome WHtR of 25% in Greenland. The equivalent in Ukraine was 0.6%. In Greenland, 20% had missing BMI *z-*score, whereas the equivalent in Ukraine was 0.2%.

**Table 1:** Characteristics of mothers and their children [*n* (%) or median (10th–90th percentile)].

Characteristic	Greenland (*n *= 531)	Ukraine (*n *= 491)
Child characteristics
Sex
Male	286 (54)	260 (53)
Female	242 (46)	228 (47)
Missing (*n*)	3	3
Gestational age (weeks)
≥ 37	503 (95)	479 (98)
< 37	25 (5)	9 (2)
Missing (*n*)	3	3
Gestational age at blood sampling	25 (5–42)	23 (9–40)
Missing (*n*)	3	3
Birth weight (g)	3,600 (2,840–4,370)	3,285 (2,800–3,800)
Missing (*n*)	6	1
Age at follow-up (years)	8.3 (7.3–9.1)	7.0 (6.6–7.6)
Missing (*n*)	10	0
Anthropometric measures at follow-up
Weight (kg)	29.0 (23.7–38.3)	24.0 (20.0–30.0)
Missing (*n*)	93	0
Height (cm)	131 (122–138)	124 (117–131)
Missing (*n*)	45	0
BMI, kg/m^2^	17.0 (14.9–20.6)	15.8 (13.9–18.3)
Missing (*n*)	98	0
BMI *z-*scores	0.7 (–0.5–2.2)	0.2 (–1–1.5)
Missing (*n*)	105	3
Waist-to-height ratio	0.5 (0.4–0.6)	0.4 (0.4–0.5)
Waist-to-height ratio > 0.5	124 (31)	35 (7)
Missing (*n*)	136	1
Weight status
Normal (≥ –2 to ≤ 1 SD)	272 (64)	381 (78)
Overweight (+1 SD)	103 (24)	66 (13)
Obese (+2 SD)	49 (11)	29 (6)
Underweight (< –2 SD)	2 (1)	14 (3)
Missing (*n*)	105	1
Maternal characteristics
Maternal age at birth (years)	26 (20–36)	24 (20–32)
Missing (*n*)	38	24
Maternal prepregnancy BMI (kg/m^2^)	24 (20–30)	21 (18–26)
Missing (*n*)	2	8
Parity
0	169 (32)	399 (81)
> 0	362 (68)	92 (19)
Maternal smoking during pregnancy
Yes (serum cotinine > 10 ng/mL)	297 (56)	76 (15)
No (serum cotinine ≤ 10 ng/mL)	234 (44)	415 (85)
Maternal educational level
Unskilled	226 (43)	183 (37)
Skilled/professional	305 (47)	308 (63)
BMI, body mass index. There are no missing data for parity, maternal smoking during pregnancy, or maternal education.		

The country-specific medians, ranges, and tertiles of PFOA and PFOS are presented in Table 2. Median exposure levels were highest in Greenland with a median (range) PFOA serum concentration of 1.8 (0.5–5.1) ng/mL, and a PFOS concentration of 20.2 (4.1–87.3) ng/mL; in comparison, exposure levels in Ukraine were for PFOA 1.0 (0.2–9.8) ng/mL, and for PFOS 5.0 (0.7–18.1) ng/mL.

**Table 2:** Description of pregnancy PFOA and PFOS (ng/mL): medians (ranges) and tertiles.

Substance/country	Median (range)	Low tertile	Medium tertile	High tertile
PFOA
Greenland	1.8 (0.5–5.1)	0.5–1.5	1.5–2.2	2.2–5.1
Ukraine	1.0 (0.2–9.8)	0.2–0.8	0.8–1.1	1.1-9.8
PFOS
Greenland	20.2 (4.1–87.3)	4.1–16.8	16.8-23.9	23.9–87.3
Ukraine	5.0 (0.7–18.1)	0.7–4.2	4.2–5.9	5.9–18.1

The results of adjusted RR of overweight in relation to pregnancy levels of PFOA and PFOS are presented in Table 3. For PFOA, the overall adjusted RR of being overweight in Greenland was 1.11 [95% confidence interval (CI): 0.82, 1.53] per natural logarithm unit (ln-unit) increase. In girls, overweight was increased in the highest PFOA tertile compared with the lowest (RR = 1.81; 95% CI: 1.04, 3.17), whereas there was no association in boys (RR = 1.03; 95% CI: 0.66, 1.59; *p* for interaction = 0.15). In Ukraine, the overall risk of overweight was inconsistent in relation to prenatal PFOA exposure: The RR was 1.38 (95% CI: 0.91, 2.10) comparing medium with low PFOA–exposed children, whereas the high versus low PFOA exposed tended to be of lower risk (RR = 0.78; 95% CI: 0.47, 1.29). A similar pattern was seen among girls in Ukraine, but was not as evident in boys. In a pooled analysis of Greenlandic and Ukrainian children, the RR for a 1–ln-unit increase in PFOA was positive, but not statistically significant (RR = 1.11; 95% CI: 0.88, 1.38). No clear association was found between prenatal PFOS exposure and offspring overweight. Crude RRs of overweight in relation to PFOA and PFOS were generally similar to the adjusted RRs, although overall estimates for PFOA in Greenland were stronger (see Supplemental Material, Table S2).

**Table 3:** Maternal PFOA and PFOS concentrations during pregnancy and adjusted relative risk (RR) of offspring overweight [WHO > 85th percentile (sex and age standardized)] at 5–9 years.

Substance/country/tertile	Overall		Girls		Boys		*p*-Interaction
	*n*	Adjusted^*a*^ RR (95% CI)	*n*	Adjusted^*a*^ RR (95% CI)	*n*	Adjusted^*a*^ RR (95% CI)	Exposure × sex
PFOA
Greenland
Low	177	1.00	82	1.00	96	1.00
Medium	177	1.24 (0.89, 1.75)	82	1.56 (0.90, 2.72)	95	1.02 (0.67, 1.57)	0.10
High	177	1.23 (0.87, 1.74)	81	1.81 (1.04, 3.17)	95	1.03 (0.66, 1.59)	0.15
Continuous^*b*^	531	1.11 (0.82, 1.53)	245	1.34 (0.82, 2.19)	286	0.98 (0.66, 1.46)	0.22
Ukraine
Low	164	1.00	77	1.00	87	1.00
Medium	164	1.38 (0.91, 2.10)	77	1.29 (0.63, 2.66)	87	1.43 (0.85, 2.40)	0.90
High	163	0.78 (0.47, 1.29)	77	0.40 (0.13, 1.19)	86	1.04 (0.60, 1.80)	0.11
Continuous^*b*^	491	1.02 (0.72, 1.44)	231	0.62 (0.32, 1.20)	260	1.34 (0.89, 2.02)	0.04
Greenland & Ukraine
Continuous^*b*^^,^^*c*^	1,022	1.11 (0.88, 1.38)	476	1.07 (0.76, 1.52)	546	1.15 (0.86, 1.53)	0.31
PFOS
Greenland
Low	177	1.00	82	1.00	96	1.00
Medium	177	0.95 (0.72, 1.26)	82	1.30 (0.83, 2.03)	95	0.72 (0.48, 1.07)	0.03
High	177	0.84 (0.61, 1.14)	81	1.09 (0.66, 1.79)	95	0.75 (0.51, 1.10)	0.23
Continuous^*b*^	531	0.91 (0.69, 1.20)	245	1.15 (0.76, 1.74)	286	0.74 (0.50, 1.11)	0.15
Ukraine
Low	164	1.00	77	1.00	87	1.00
Medium	164	0.91 (0.60, 1.40)	77	0.60 (0.27, 1.33)	87	1.07 (0.64, 1.80)	0.14
High	163	0.89 (0.57, 1.37)	77	0.89 (0.43, 1.85)	86	0.85 (0.48, 1.48)	0.86
Continuous^*b*^	491	1.10 (0.75, 1.60)	231	0.84 (0.42, 1.69)	260	1.22 (0.78, 1.91)	0.22
Greenland & Ukraine
Continuous^*b*^^,^^*c*^	1,022	0.97 (0.78, 1.21)	476	1.06 (0.74, 1.51)	546	0.92 (0.69, 1.23)	0.06
^***a***^Adjusted for maternal age at birth, maternal prepregnancy body mass index, smoking during pregnancy, maternal education, and parity. ^***b***^Continuous PFOA and PFOS are natural logarithm transformed. ^***c***^Additionally adjusted for country. Multiple imputation was used to impute missing data.							

Associations between PFOA and BMI *z-*scores in Greenland were positive, but were not statistically significant after adjustment (see Supplemental Material, Table S3). In Ukraine, medium PFOA exposure was associated with BMI *z-*scores in the adjusted model (β = 0.28: 95% CI: 0.03, 0.52). No associations were observed between crude or adjusted models of PFOA and BMI *z-*score in Ukraine or between PFOS and BMI *z-*score in the two countries.

The RR of having WHtR > 0.5 in relation to prenatal PFOA and PFOS exposures is presented in Table 4. The overall RR of having WHtR > 0.5 in relation to PFOA was elevated in both countries (Greenland, overall RR = 1.28; 95% CI: 0.91, 1.82; Ukraine, overall RR = 1.50; 95% CI: 0.83, 2.72) and in the pooled analysis (pooled, overall RR = 1.30; 95% CI: 0.97, 1.74). The overall RR of having WHtR > 0.5 in relation to continuous PFOS was increased in both countries, and in the pooled analysis, the RR was 1.38 (95% CI: 1.05, 1.82). In Greenland, the overall continuous model showed a RR of 1.31 (95% CI: 0.97, 1.77) in relation to PFOS. In Ukraine, the risk was also increased (RR = 1.67; 95% CI: 0.81, 3.47).

**Table 4:** Maternal PFOA and PFOS concentrations during pregnancy and adjusted relative risk (RR) of offspring waist-to-height ratio > 0.5 at 5–9 years.

Substance/country/tertile	Overall		Girls		Boys		*p*-Interaction
	*n*	Adjusted^*a*^ RR (95% CI)	*n*	Adjusted^*a*^ RR (95% CI)	*n*	Adjusted^*a*^ RR (95% CI)	Exposure × sex
PFOA
Greenland
Low	177	1.00	82	1.00	96	1.00
Medium	177	1.32 (0.92, 1.90)	82	1.93 (1.15, 3.24)	95	0.92 (0.52, 1.61)	0.06
High	177	1.18 (0.80, 1.74)	81	1.65 (0.94, 2.89)	95	1.11 (0.65, 1.90)	0.30
Continuous^*b*^	531	1.28 (0.91, 1.82)	245	1.49 (0.95, 2.33)	286	1.10 (0.66, 1.84)	0.27
Ukraine
Low	164	1.00	77	1.00	87	1.00
Medium	164	1.33 (0.61, 2.89)	77	4.14 (0.81, 21.29)	87	0.80 (0.27, 2.34)	0.11
High	163	1.11 (0.48, 2.57)	77	1.04 (0.12, 8.98)	86	1.24 (0.48, 3.22)	0.89
Continuous^*b*^	491	1.50 (0.83, 2.72)	231	1.06 (0.44, 2.55)	260	1.71 (0.74, 3.92)	0.65
Greenland & Ukraine
Continuous^*b*^^,^^*c*^	1,022	1.30 (0.97, 1.74)	476	1.41 (0.97, 2.05)	546	1.25 (0.80, 1.95)	0.06
PFOS
Greenland
Low	177	1.00	82	1.00	96	1.00
Medium	177	1.14 (0.80, 1.63)	82	1.64 (1.01, 2.66)	95	0.83 (0.48, 1.43)	0.08
High	177	1.22 (0.86, 1.74)	81	1.63 (0.99, 2.68)	95	1.06 (0.63, 1.77)	0.30
Continuous^*b*^	531	1.31 (0.97, 1.77)	245	1.44 (0.98, 2.11)	286	1.19 (0.72, 1.97)	0.57
Ukraine
Low	164	1.00	77	1.00	87	1.00
Medium	164	1.43 (0.63, 3.25)	77	0.55 (0.10, 2.99)	87	1.85 (0.72, 4.80)	0.32
High	163	1.44 (0.62, 3.31)	77	1.78 (0.44, 7.18)	86	0.98 (0.33, 2.92)	0.41
Continuous^*b*^	491	1.67 (0.81, 3.47)	231	2.45 (0.62, 9.60)	260	1.36 (0.57, 3.26)	0.33
Greenland & Ukraine
Continuous^*b*^^,^^*c*^	1,022	1.38 (1.05, 1.82)	476	1.54 (1.06, 2.23)	546	1.24 (0.82, 1.87)	0.01
WHtR, waist-to-height-ratio. ^***a***^Adjusted for child sex, child age at follow-up, maternal age at birth, maternal prepregnancy body mass index, smoking during pregnancy, maternal education, and parity. ^***b***^Continuous PFOA and PFOS are natural logarithm transformed. ^***c***^Additionally adjusted for country. Multiple imputation was used to impute missing data.							

When stratified by sex, Greenlandic girls tended to have a higher RR of having WHtR > 0.5 in relation to PFOS than boys (Table 4). Interaction was evident in the pooled estimate of Greenland and Ukraine (*p* for interaction between 0.01 and 0.06). The overall crude RRs were in the same direction (see Supplemental Material, Table S2). There was no clear trend according to age of the child at follow-up, and the *p*-value for test of interaction between exposures and age (dichotomized at median age) at follow-up was between 0.13 and 0.47 in pooled analysis (data not shown).

PFOA in relation to continuous WHtR showed weak positive associations in crude and adjusted models in Greenland (adjusted β = 0.01; 95% CI: 0.00, 0.02) (see Supplemental Material, Table S3). No clear associations were observed in Ukraine in relation to PFOA or PFOS.

Including both pollutants in the model did not change results notably (data not shown).

Restricting analysis to children of normal birth weight, adjusting for gestational age at blood sampling, and including sugar intake, fruit and vegetable intake, and physical activity of the child, generally showed results similar to the main analysis (data not shown). Comparing complete case results with imputation-based results showed similar results (see Supplemental Material, Table S4). In the complete case results of Greenlandic and Ukrainian children, the RR of having WHtR > 0.5 in relation to continuous PFOS exposure was 1.54 (95% CI: 1.16, 2.06), whereas the imputation-based results of the same RR was 1.38 (95% CI: 1.05, 1.82). Finally, results did not materially change when we used 20 or 150 data sets (instead of 100) for the imputation-based model (data not shown).

## Discussion

Higher prenatal exposure to PFOA and PFOS was associated with increased prevalence of WHtR > 0.5 in a pooled analysis of Greenlandic and Ukrainian children of 5–9 years of age. RRs for WHtR > 0.5 in association with a 1-unit increase in ln PFOA and PFOS were somewhat larger but less precise for Ukraine than Greenland [RR = 1.50 (95% CI: 0.83, 2.72) vs. RR = 1.28 (95% CI: 0.91, 1.82) for PFOA; RR = 1.67 (95% CI: 0.81, 3.47) vs. RR = 1.31 (95% CI: 0.97, 1.77) for PFOS]. Little or no association was observed for prenatal PFOA and PFOS exposure and risk of overweight.

Suggested mechanisms that could explain the association between PFAS and WHtR are not well established. However, animal studies suggest that PFAS may act as endocrine disruptors affecting estrogen concentrations in the body (Shi et al. 2009); but also, effects have been reported through an activation of peroxisome proliferator–activated receptor alpha (PPARα) that interacts with liver estrogen receptor (Rosen et al. 2008; Wolf et al. 2008). Two experimental rat studies have found that half-life of the PFAS differs according to sex (Ohmori et al. 2003; Vanden Heuvel et al. 1991); furthermore, multiple studies have reported that endocrine disruptors could change the metabolic pathways and cause permanent changes in body weight as reviewed by Newbold et al. (2008).

In one study, Halldorsson et al. (2012) reported a modest positive association between prenatal PFOA and overweight and central obesity (i.e., waist circumference) in young female adults (mean age, 20 years); we also found consistent indications of this among the girls in Greenland, but our findings were not consistent between countries. The lack of a statistically significant association among the girls in Ukraine in our study compared with the aforementioned study could be attributable to lower PFOA levels in Ukraine (median, 1.0 ng/mL) in the current study versus 3.7 ng/mL in the study by Halldorsson et al. (2012). Further, only approximately 5% of the girls in Ukraine had WHtR > 0.5 (data not shown), which makes our sex-stratified results uncertain.

It is possible that the sensitivity of the measure of childhood adiposity based on BMI *z-*score cut-offs is not as good as that of the WHtR. WHtR has been suggested to be equal or superior to BMI as a marker for cardiometabolic morbidity later in life in children between 4 and 18 years, because children within the same BMI category but with larger waist circumference had higher risk of having metabolic syndrome, high diastolic blood pressure, and elevated insulin level (Maffeis et al. 2008; Mokha et al. 2010). However, there is not a general consensus on this (Blüher et al. 2013). The long-term consequences of metabolic syndrome, high diastolic blood pressure, and elevated insulin level are related to serious sequelae and early death. Another study investigating the association between prenatal exposure to PFAS and offspring overweight reported no association between prenatal PFOA [median, 5.25 ng/L (interquartile range, 0.5, 21.9 ng/L)] at gestational week 8 (interquartile range, 3–15) and BMI *z-*scores and overweight at age 7 years in a random sample (*n* = 811) from the Danish National Birth Cohort (DNBC) (Andersen et al. 2013). Their null finding on PFOA and overweight is in line with our study, but it is not consistent with the aforementioned study on 20-year-old females (Halldorsson et al. 2012).

We found no association between PFOS and overweight, which is consistent with both prior studies (Andersen et al. 2013; Halldorsson et al. 2012).

In the study by Andersen et al. (2013), the authors reported no associations between prenatal PFOA and PFOS exposures and residuals of WHtR, but no estimates were reported. In our study, PFOA and PFOS were associated with WHtR > 0.5 in Greenland and Ukraine, especially among girls. The differences according to sex were also reported by Halldorsson et al. (2012) in relation to overweight and waist circumference and may in part relate to possible changes in estrogen levels in relation to the compounds (Halldorsson et al. 2012; Shi et al. 2009). In line with this, our pooled analysis of Greenland and Ukraine indicated an interaction between PFOS and sex. Hence, the sex-stratified results of the pooled analysis of the Greenlandic and Ukrainian populations is the most reliable compared with the overall results.

This study has some limitations. Children were weighed using different weighing scales and height measures at the examination. It is possible that the true association would be stronger if the measuring equipment had been the same. In Ukraine, using standardized measurements the strongest associations to WHtR were observed; but possibly due to lack of power, the country-stratified analyses were not statistically significant. Although the misclassification is believed to be stronger in Greenland, we believe that any misclassification is nondifferential. Also, the missing WHtR of 25% of the study population in Greenland could possibly introduce information bias. However, we believe any misclassification would be nondifferential although the direction of bias is uncertain.

Also, levels of PFAS tend to decrease during pregnancy (Fei et al. 2007) and, because blood samples in the present study were collected throughout pregnancy, there is a risk of exposure misclassification. In a sensitivity analysis, we adjusted for gestational age at blood sampling, which did not change the results (data not shown); thus, gestational age at blood sampling does not seem to be of major concern.

Supplementary analyses of our data stratified by median age indicated no clear difference in associations according to age (data not shown), and also the test for effect modification by age group was statistically nonsignificant. Furthermore, stratification induces loss of power and hence should preferably be addressed in a larger cohort of children.

Strengths of the study include the prospective follow-up through 9 years in birth cohorts from the early 2000s when PFAS levels around the world were very high. The study population including mother–child pairs from a European and an Arctic area enabled evaluation of consistency of exposure–outcome association across ethnicity and regions.

## Conclusions

Our results indicate that prenatal PFOA and PFOS exposure may be associated with child obesity around the waist (WHtR > 0.5), but not unequivocally with overweight at 5–9 years. There were some indications that females may be more sensitive to exposure than males. The implications of adiposity around the waist are related to higher risk of having metabolic syndrome, high diastolic blood pressure, and high insulin level, and are of great concern because one-third of the children in Greenland had a WHtR > 5.

## Supplemental Material

(299 KB) PDF
