# Supplementary material for: Anthropometry in 5- to 9-Year-Old Greenlandic and Ukrainian Children in Relation to Prenatal Exposure to Perfluorinated Alkyl Substances
Source: Environ Health Perspect. 2015 Mar 26;123(8):841–6. doi: 10.1289/ehp.1408881 (PMC4529015; doi:10.1289/ehp.1408881)
Supplement: (299 KB) PDF [file ehp.1408881.s001.acco.pdf]

Environ Health Perspect
doi: 10.1289/ehp.1408881

Note to Readers: ***EHP*** **strives to ensure that all journal content is accessible to all readers. However, some figures and Supplemental Material published in *EHP* articles may not conform to 508 standards due to the complexity of the information being presented. If you need assistance accessing journal content, please contact ehp508@niehs.nih.gov. Our staff will work with you to assess and meet your accessibility needs within 3 working days.**

Supplemental Material

Anthropometry in 5- to 9-Year-Old Greenlandic and Ukrainian Children in Relation to Prenatal Exposure to Perfluorinated Alkyl Substances

Birgit Bjerre Høyer, Cecilia Høst Ramlau-Hansen, Martine Vrijheid, Damaskini Valvi, Henning Sloth Pedersen, Valentyna Zviezdai, Bo A.G. Jönsson, Christian H. Lindh, Jens Peter Bonde, and Gunnar Toft

Table of Contents

Table S1. Characteristics of participants and those lost to follow-up.

Table S2. Maternal PFOA and PFOS concentrations during pregnancy and crude relative risk (RR) of offspring overweight (WHO > 85th percentile (age and sex standardised)) and waist-to-height-ratio >0.5 at 5 to 9 years.

Table S3. Maternal PFOA and PFOS concentrations during pregnancy and crude and adjusted associations with BMI z-scores (sex and age standardised) and WHtR in offspring at 5 to 9 years.

Table S4. Complete-case-analysis of maternal PFOA and PFOS concentrations during pregnancy and crude and adjusted relative risk (RR) of offspring overweight (WHO > 85th percentile (sex and age standardised)) and waist-height-ratio >0.5 at 5 to 9 years.

Table S1. Characteristics of participants and those lost to follow-up.

Characteristic	Lost to follow-up[a] Greenland (n=40)	Participants[b] Greenland (n=531)	Lost to follow-up[c] Ukraine (n=119)	Participants[d] Ukraine (n=491)
**PFOA,** ng/mL [Median (range)]	1.7 (0.5—3.3)	1.8 (0.5—5.1)	0.9 (0.3—2.6)	1.0 (0.2—9.8)
**PFOS,** ng/mL [Median (10th 90th percentile)]	19.4 (10.9—67.7)	20.2 (4.1—87.3)	5.1 (0.8—13.3)	5.0 (15.0—37.9)
**Maternal pre-pregnancy BMI,** kg/m^2 [Mean (SD)]	24.2 (4.6)	24.5 (4.5)	21.6 (2.9)	21.7 (3.3)
**Paternal BMI**, kg/m^2 [Mean (SD)]	25.8 (3.4)	26.8 (3.8)	24.3 (3.2)	24.2 (2.9)

Abbreviations: BMI, body mass index; PFOA, perfluorooctanoate; PFOS, perfluorooctane sulfonate.

[a]In the lost to follow-up group, 27.5% had missing information on paternal BMI. [b]Among the participants, 0.4% had missing information on maternal BMI and 25.0% had missing information on paternal BMI. [c]In the lost to follow-up group, 0.8% had missing PFOA, PFOS and maternal BMI and 4.2% had missing paternal BMI. [d]Among the participants, 1.6% had missing information on maternal BMI and 3.3% had missing information on paternal BMI.

Table S2. Maternal PFOA and PFOS concentrations during pregnancy and crude relative risk (RR) of offspring overweight (WHO > 85th percentile (age and sex standardised)) and waist-to-height-ratio >0.5 at 5 to 9 years.

Exposure	Level	Overweight		WHtR >0.5	
		n	Crude RR (95 % CI)	n	Crude RR (95 % CI)
**Greenland**					
PFOA	Low	177	1.00	177	1.00
	Medium	177	1.47 (1.07, 2.02)	177	1.34 (0.95, 1.89)
	High	177	1.41 (1.02, 1.94)	177	1.12 (0.78, 1.59)
	Continuous[a]	531	1.31 (0.99, 1.72)	531	1.21 (0.90, 1.63)
PFOS	Low	177	1.00	177	1.00
	Medium	177	1.04 (0.78, 1.38)	177	1.12 (0.79, 1.59)
	High	177	0.89 (0.65, 1.22)	177	1.19 (0.85, 1.68)
	Continuous[a]	531	0.95 (0.72, 1.24)	531	1.26 (0.94, 1.68)
**Ukraine**					
PFOA	Low	164	1.00	164	1.00
	Medium	164	1.34 (0.90, 2.04)	164	1.18 (0.54, 2.56)
	High	163	0.78 (0.48, 1.27)	163	1.01 (0.45, 2.27)
	Continuous[a]	491	1.02 (0.73, 1.42)	491	1.36 (0.79, 2.32)
PFOS	Low	164	1.00	164	1.00
	Medium	164	0.92 (0.60, 1.40)	164	1.45 (0.64, 3.31)
	High	163	0.86 (0.56, 1.34)	163	1.45 (0.64, 3.31)
	Continuous[a]	491	1.07 (0.74, 1.56)	491	1.70 (0.87, 3.32)

Abbreviations: CI, confidence interval; PFOA, perfluorooctanoate; PFOS, perfluorooctane sulfonate; RR, relative risk; WHtR, waist-to-height-ratio.

[a]Continuous PFOA and PFOS are natural logarithm transformed.

Multiple imputation was used to impute missing data.

Table S3. Maternal PFOA and PFOS concentrations during pregnancy and crude and adjusted associations with BMI z-scores (sex and age standardised) and WHtR in offspring at 5 to 9 years.

Exposure	Level	n	BMI z-score Crude β (95 % CI)	BMI z-score Adjusted[a] β (95% CI)	n	WHtR Crude β (95 % CI)	WHtR Adjusted[b] β (95 % CI)
**PFOA**							
Greenland	Low	177	reference	reference	177	reference	reference
	Medium	177	0.23 (-0.03, 0.49)	0.09 (-0.18, 0.35)	177	0.01 (0.00, 0.02)*	0.01 (0.00, 0.02)
	High	177	0.30 (0.05, 0.56)*	0.19 (-0.09, 0.46)	177	0.01 (0.00, 0.02)	0.01 (0.00, 0.02)
	Continuous[c]	531	0.25 (0.01, 0.49)**	0.11 (-0.14, 0.37)	531	0.01 (0.00, 0.02)**	0.01 (0.00, 0.02)**
Ukraine	Low	164	reference	reference	164	reference	reference
	Medium	164	0.21 (-0.03, 0.46)	0.28 (0.03, 0.52)*	164	0.00 (-0.01, 0.01)	0.00 (-0.01, 0.01)
	High	163	-0.01 (-0.25, 0.24)	0.07 (-0.18, 0.32)	163	0.00 (-0.01, 0.01)	0.00 (-0.01, 0.01)
	Continuous[c]	491	0.03 (-0.17, 0.23)	0.02 (-0.19, 0.23)	491	0.00 (-0.01, 0.01)	0.00 (-0.01, 0.01)
**PFOS**							
Greenland	Low	177	reference	reference	177	reference	reference
	Medium	177	0.00 (-0.25, 0.26)	-0.06 (-0.31, 0.19)	177	0.01 (0.00, 0.02)	0.01 (0.00, 0.02)
	High	177	-0.11 (-0.37, 0.15)	-0.15 (-0.41, 0.10)	177	0.01 (-0.01, 0.02)	0.00 (0.00, 0.02)
	Continuous[c]	531	-0.05 (-0.28, 0.19)	-0.07 (-0.31, 0.16)	531	0.00 (0.00, 0.01)	0.00 (0.00, 0.02)
Ukraine	Low	164	reference	reference	164	reference	reference
	Medium	164	-0.01 (-0.25, 0.23)	-0.03 (-0.27, 0.22)	164	0.00 (-0.01, 0.01)	0.00 (-0.01, 0.01)
	High	163	-0.01 (-0.25, 0.24)	0.03 (-0.22, 0.28)	163	0.01 (0.00, 0.02)*	0.01 (0.00, 0.02)
	Continuous[c]	491	0.03 (-0.19, 0.25)	0.04 (-0.19, 0.26)	491	-0.01 (-0.02, 0.01)	0.00 (0.00, 0.01)

Abbreviations: CI, confidence interval; PFOA, perfluorooctanoate; PFOS, perfluorooctane sulfonate; WHtR, waist-to-height-ratio.

[a]Adjusted for maternal age at birth, maternal pre-pregnancy body mass index, smoking during pregnancy, maternal education and parity. [b]Adjusted for child sex, child age at follow-up, maternal age at birth, maternal pre-pregnancy body mass index, smoking during pregnancy, maternal education and parity. [c]Continuous PFOA and PFOS are natural logarithm transformed and β is the change in outcome for one natural log-unit of exposure.

*Statistically significantly different compared to low exposed children, $p < 0.05$.

**Statistically significantly different from 0 for one natural log-unit increase in exposure, $p < 0.05$.

Multiple imputation was used to impute missing data.

Table S4. Complete-case-analysis of maternal PFOA and PFOS concentrations during pregnancy and crude and adjusted relative risk (RR) of offspring overweight (WHO > 85th percentile (sex and age standardised)) and waist-height-ratio >0.5 at 5 to 9 years.

Exposure	Level	Cases/n	Overweight Crude RR (95% CI)	Cases/n	Overweight Adjusted[b] RR (95% CI)	Cases/n	WHtR >0.5 Crude RR (95% CI)	Cases/n	WHtR >0.5 Adjusted[c] RR (95 % CI)
**PFOA**									
Greenland	Low	38/143	1.00	35/135	1.00	35/130	1.00	43/117	1.00
	Medium	57/136	1.57 (1.12, 2.19)	55/127	1.40 (0.98, 2.01)	49/126	1.44 (1.01, 2.07)	36/114	1.50 (1.02, 2.21)
	High	57/147	1.47 (1.05, 2.06)	53/133	1.27 (0.88, 1.84)	40/139	1.07 (0.73, 1.57)	37/122	1.20 (0.80, 1.82)
	Continuous[a]	152/426	1.35 (1.01, 1.79)	143/395	1.13 (0.82, 1.57)	124/395	1.16 (0.85, 1.57)	116/353	1.28 (0.90, 1.82)
Ukraine	Low	31/164	1.00	30/155	1.00	11/164	1.00	10/155	1.00
	Medium	40/161	1.31 (0.87, 1.99)	39/150	1.37 (0.89, 2.10)	13/164	1.18 (0.55, 2.56)	12/150	1.41 (0.62, 3.21)
	High	24/163	0.81 (0.50, 1.31)	23/156	0.79 (0.48, 1.32)	11/162	1.01 (0.45, 2.27)	10/155	1.13 (0.47, 2.72)
	Continuous[a]	95/488	1.04 (0.75, 1.45)	92/461	1.00 (0.70, 1.41)	35/490	1.36 (0.79, 2.32)	32/460	1.43 (0.78, 2.63)
Greenland & Ukraine[d]	Continuous[a]	247/914	1.20 (0.97, 1.48)	235/856	1.12 (0.90, 1.41)	159/885	1.21 (0.92, 1.58)	148/813	1.29 (0.96, 1.75)
**PFOS**									
Greenland	Low	51/139	1.00	48/132	1.00	32/122	1.00	32/112	1.00
	Medium	55/143	1.05 (0.78, 1.42)	51/132	0.93 (0.69, 1.27)	42/130	1.23 (0.84, 1.82)	38/116	1.18 (0.80, 1.74)
	High	46/144	0.87 (0.63, 1.20)	44/131	0.85 (0.61, 1.18)	50/143	1.33 (0.92, 1.94)	46/125	1.31 (0.90, 1.92)
	Continuous[a]	152/426	0.96 (0.73, 1.27)	143/395	0.93 (0.79, 1.25)	124/395	1.35 (1.00, 1.83)	116/353	1.47 (1.08, 2.00)
Ukraine	Low	35/164	1.00	34/153	1.00	9/164	1.00	8/153	1.00
	Medium	31/163	0.89 (0.58, 1.37)	31/157	0.89 (0.58, 1.37)	13/163	1.45 (0.64, 3.31)	13/156	1.66 (0.71, 3.87)
	High	29/161	0.87 (0.56, 1.35)	27/151	0.86 (0.55, 1.34)	13/163	1.45 (0.64, 3.31)	11/151	1.42 (0.58, 3.46)
	Continuous[a]	95/488	1.08 (0.74, 1.58)	92/461	1.09 (0.74, 1.59)	35/490	1.70 (0.87, 3.31)	32/460	1.67 (0.80, 3.47)
Greenland & Ukraine[d]	Continuous[a]	247/914	1.01 (0.80, 1.26)	235/856	1.00 (0.79, 1.26)	159/885	1.42 (1.08, 1.87)	148/813	1.54 (1.16, 2.06)

Abbreviations: CI, confidence interval; PFOA, perfluorooctanoate; PFOS, perfluorooctane sulfonate; RR, relative risk; WHtR, waist-to-height-ratio.

[a]Continuous PFOA and PFOS are natural logarithm transformed. [b]Adjusted for maternal age at birth, maternal pre-pregnancy body mass index, smoking during pregnancy, maternal education and parity. [c]Adjusted for child sex, age at follow-up, maternal age at birth, maternal pre-pregnancy body mass index, smoking during pregnancy, maternal education and parity. [d]In addition adjusted for country.
